# The spatial distribution and biogeochemical drivers of nitrogen cycle genes in an Antarctic desert

**DOI:** 10.3389/fmicb.2022.927129

**Published:** 2022-10-06

**Authors:** Francisco Pascoal, Inês Areosa, Luís Torgo, Paula Branco, Mafalda S. Baptista, Charles K. Lee, S. Craig Cary, Catarina Magalhães

**Affiliations:** ^1^Interdisciplinary Center of Marine and Environmental Research, University of Porto, Porto, Portugal; ^2^Faculty of Sciences, University of Porto, Porto, Portugal; ^3^Faculty of Computer Science, Dalhousie University, Halifax, NS, Canada; ^4^School of Electrical Engineering and Computer Science, University of Ottawa, Ottawa, ON, Canada; ^5^International Centre for Terrestrial Antarctic Research, University of Waikato, Hamilton, New Zealand; ^6^School of Science, University of Waikato, Hamilton, New Zealand

**Keywords:** nitrogen cycle, Antarctic desert, Antarctic metagenomics, nitrogen-cycling genes, association rules, McMurdo Dry Valleys

## Abstract

Antarctic deserts, such as the McMurdo Dry Valleys (MDV), represent extremely cold and dry environments. Consequently, MDV are suitable for studying the environment limits on the cycling of key elements that are necessary for life, like nitrogen. The spatial distribution and biogeochemical drivers of nitrogen-cycling pathways remain elusive in the Antarctic deserts because most studies focus on specific nitrogen-cycling genes and/or organisms. In this study, we analyzed metagenome and relevant environmental data of 32 MDV soils to generate a complete picture of the nitrogen-cycling potential in MDV microbial communities and advance our knowledge of the complexity and distribution of nitrogen biogeochemistry in these harsh environments. We found evidence of nitrogen-cycling genes potentially capable of fully oxidizing and reducing molecular nitrogen, despite the inhospitable conditions of MDV. Strong positive correlations were identified between genes involved in nitrogen cycling. Clear relationships between nitrogen-cycling pathways and environmental parameters also indicate abiotic and biotic variables, like pH, water availability, and biological complexity that collectively impose limits on the distribution of nitrogen-cycling genes. Accordingly, the spatial distribution of nitrogen-cycling genes was more concentrated near the lakes and glaciers. Association rules revealed non-linear correlations between complex combinations of environmental variables and nitrogen-cycling genes. Association rules for the presence of denitrification genes presented a distinct combination of environmental variables from the remaining nitrogen-cycling genes. This study contributes to an integrative picture of the nitrogen-cycling potential in MDV.

## Introduction

The McMurdo Dry Valleys (MDV), largely snow-free valleys in Antarctica, is one of the most inhospitable ecosystems on earth, because of their oligotrophic nature, low water availability, high pH and salinity, and low temperature. Prokaryotes are ubiquitous in this environment, but generally have low abundance and diversity (Cary et al., 2010). More complex forms of life (e.g., nematodes) are restricted to sites where prokaryotic communities are well-established and higher carbon-to-nitrogen ratio is observed (Wood et al., 2008; Magalhães et al., 2012). The low water availability, high pH, and low nutrient content (including low nitrogen) are involved in shaping a taxonomically and functionally unique soil microbiome in MDV (Fierer et al., 2012). By approaching the cold, dry and oligotrophic limits of life (Goordial et al., 2016), MDV represents a natural laboratory to explore the biogeochemical requirements of life (McKay, 2014).

Considering how limited nitrogen is in MDV, as previously shown by its very low nitrogen to phosphorus to carbon stoichiometry (Barrett et al., 2007), different studies have surveyed the biogeochemical potential of nitrogen-cycling in this extreme environment, extensively reviewed elsewhere (Ortiz et al., 2020). One example of nitrogen relevance was given in a study from glacial melt water stream, where the addition of a nitrogen source *in situ* increased unicellular producers’ abundance and the combination of a nitrogen and phosphorous source increased its diversity (Kohler et al., 2016).

Current understanding of the microbial nitrogen cycle at the genetic level has been thoroughly reviewed elsewhere (Kuypers et al., 2018). The cycle begins with molecular nitrogen fixation to ammonia (*N*_2_ to *NH*_3_), that can be assimilated into organic nitrogen (for biomass) or can undergo the process of nitrification (*NH*_3_ to *NO_3_^–^*). Nitrate can then be reduced back to ammonia (*NO_3_^–^* to *NH*_3_) for energy (Dissimilatory Nitrate Reduction–DNR), or it can be used for biosynthesis (Assimilatory Nitrate Reduction–ANR). Nitrite can also be reduced to molecular nitrogen by the process of denitrification (*NO_2_^–^* to *N*_2_). Finally, the anammox reactions allow the transformation of nitric oxide and ammonia back to molecular nitrogen, using nitrite as the electron acceptor (*NO_2_^–^* and *NH*_3_ to *N*_2_).

Nitrogen fixation in Antarctic deserts was firstly identified in permanently ice-covered lakes, using *nifH* as a marker gene (Olson et al., 1998). Because nitrogen fixation was identified in both light and dark regimes, Olson et al. (1998) suggested that this environment includes auto- and heterotrophic microorganisms. Other studies have identified the *nifH* gene in soil, rocks, and hypolith environments from MDV and confirmed that it belonged to both auto and heterotrophs microorganisms (Jung et al., 2011; Chan et al., 2013; Wei et al., 2016; Coyne et al., 2020). A common autotrophic diazotroph in MDV is cyanobacteria (Jungblut and Neilan, 2010), which is not necessarily responsible for net nitrogen fixation in this environment (Niederberger et al., 2012; Lacap-Bugler et al., 2017). The co-occurrence of cyanobacteria and higher levels of diversity for heterotrophic diazotrophs supports the view that cyanobacteria are an important source of organic carbon for heterotrophic nitrogen fixers (Niederberger et al., 2012). Nitrogen fixation appears to be more determined by the complexity of inter-specific biological interactions, than the specific abiotic limitations of water availability or temperature (Coyne et al., 2020).

Nitrification studies in MDV focused mainly on the identification of the genes that encode Ammonia Monooxygenase (AMO) (Yergeau et al., 2007; Jung et al., 2011; Chan et al., 2013; Magalhães et al., 2014; Tolar et al., 2016; Wei et al., 2016; Monteiro et al., 2020). A study demonstrated that Ammonia Oxidizing Archaea (AOA) and Bacteria (AOB) have a dichotomous distribution in MDV (Magalhães et al., 2014), hinting at possible geochemical variables constraining each group differently (Jung et al., 2011). This was supported by surveys of ammonia oxidizers on other Antarctic environments, such as inland waters (Voytek et al., 1999), coastal waters (Tolar et al., 2016), and soils (Ayton et al., 2010). A microcosm from MDV soil samples found: (i) copper to be more toxic to AOB communities than AOA; (ii) electrical conductivity influenced both communities in similar ways; and (iii) organic carbon increased AOA, while it decreases AOB frequency of occurrence (Monteiro et al., 2020). The finding is coherent with the reported utilization of urea (organic carbon source) by AOA to boost nitrification in marine polar environments (Alonso-Sáez et al., 2012).

Metagenomic surveys have identified DNR-associated genes in both soil and hypolithic (under rock) samples from MDV (Chan et al., 2013; Wei et al., 2016), but with limited information on the genetic diversity or environmental variables determining the presence of DNR genes. Another study used the marker gene *nirG* for nitrate reduction (Jung et al., 2011), which may hint the presence of DNR in MDV (Calderoli et al., 2018), although its biogeochemical drivers are still unknown.

Molecular surveys in MDV have identified genes for the entire denitrification process (Yergeau et al., 2007; Jung et al., 2011; Chan et al., 2013). In MDV, *nirS* genes were consistently more abundant than *nirK* (Jung et al., 2011) and both genes increased after nitrogen addition. On the other hand, warming is another factor that lead to abundance difference between *nirK and* nirS because nirK-type denitrifiers were more sensitive to warming (Jung et al., 2011). If denitrification is fully present in MDV, then favorable conditions to denitrification can favor net nitrogen loss (Chan et al., 2013). In fact, denitrification genes were previously positively correlated with soil temperature in MDV (Yergeau et al., 2007). Because incomplete denitrification can lead to accumulation of nitrous oxide, a powerful greenhouse gas, it is relevant to understand if the MDV biological communities can carry the entire denitrification pathway and how it balances with dissimilatory and assimilatory nitrate reduction, in the context of the MDV nitrogen biogeochemistry. In fact, assimilatory nitrate reduction genes have also been identified in MDV (Wei et al., 2016), which were more abundant in soil than in hypolithic microbial communities. However, in another study, ANR genes were more prevalent in chasmoendoliths (microorganisms in rock cleft), cryptoendoliths (microorganisms within rock pores), and hypoliths (Chan et al., 2013).

Genes for anammox reactions have seldom been found in MDV. One survey identified anammox genes from Planctomycetes, with higher abundance in hypoliths than in soil samples (Wei et al., 2016) and another study identified anammox genes in both Planctomycetes and Betaproteobacteria (Chan et al., 2013).

**FIGURE 1:**
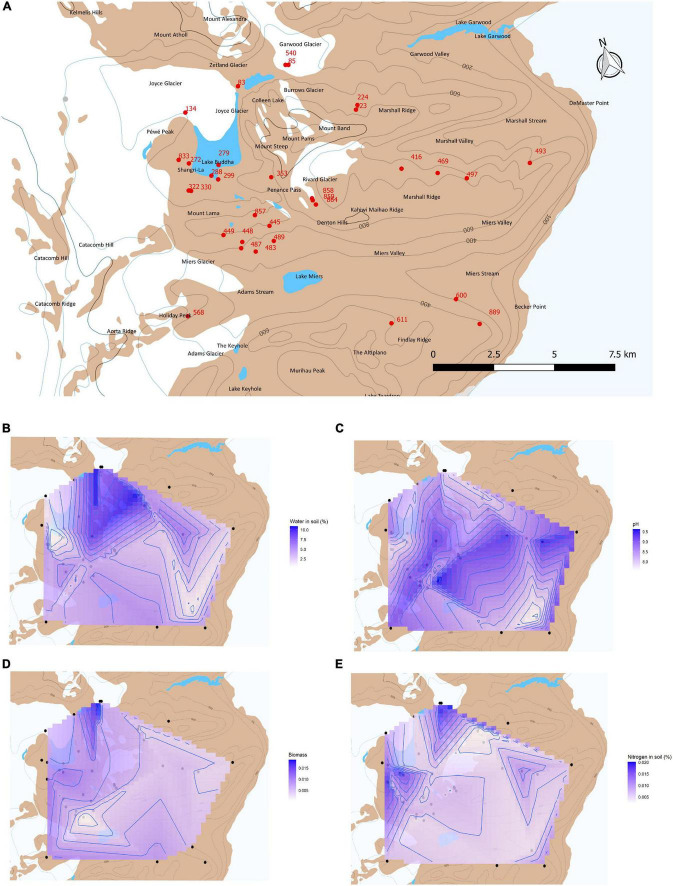
**(A)** Map with selected sampling sites (red dots) of the MDV soils used for metagenomic analyses and respective unique codes. Blue denotes lake and white denotes glacier areas. Additional maps include linear interpolation in the area between samples for **(B)** percentage of water in soil, **(C)** pH, **(D)** biomass, and **(E)** percentage of nitrogen.

The genetic potential for the pathways of the nitrogen biogeochemical cycle was previously identified (Yergeau et al., 2007; Jung et al., 2011; Chan et al., 2013; Magalhães et al., 2014; Nelson et al., 2016; Wei et al., 2016), but relevant questions remain unanswered on how the combined nitrogen pathways are spatially distributed in MDV and on how the biogeochemical variables determine its dynamics and limit its distribution. This missing piece of information is important, because it hints at the drivers controlling the nitrogen-cycling genes of MDV. Furthermore, it is necessary to integrate the perspective of genes implicated in all nitrogen-cycling processes, to understand the potential viability of the nitrogen-cycling pathways and how they interact. We hypothesized that the multiple nitrogen-cycling pathways in MDV are near the limits of their distribution and the strong physicochemical heterogeneity of MDV soils are the main selective forces that determine the complexity of the nitrogen metabolism in these environments. Here we used metagenomics and biogeochemical data of 32 MDV sites (Lee et al., 2019; Figure 1 and Supplementary Table 1), to explore, with an unprecedented integral view, all nitrogen-cycling pathways, without the constraints of focusing on single genetic markers (Calderoli et al., 2018; Ortiz et al., 2020). With this approach, we provide a detailed description of the relationships between the genes involved in the entire nitrogen-cycling and its relation with MDV spatial biogeochemistry.

## Materials and methods

The MDV soil samples collection and biogeochemical description are available in Lee et al. (2019). Our study selected the samples (*n* = 32) whose community DNA was successfully extracted and sequenced, with full metagenomes (Supplementary Table 1) and with available metadata on biogeochemical variables (Lee et al., 2019; Supplementary Table 2). Overall, the metagenomes were collected from a wide spatial range of the MDV, with most samples near the lake Buddha and the Miers glacier, several other samples were collected near the Findlay ridge, the Marshal ridge and south of Garwood glacier and valley (Figure 1). Each one of the 32 samples corresponds to a single sampling site.

The metagenomes were generated by the Joint Genome Institute, Integrated Microbial Genomes and Microbiomes (JGI IMG/M) (Chen et al., 2021; Mukherjee et al., 2021), where they are publicly available under their respective metagenome ID (Supplementary Table 1).

### Utilization of Kyoto encyclopedia of genes database, with joint genome institute, to survey nitrogen-cycling genes

The JGI IMG/M platform (Chen et al., 2021; Mukherjee et al., 2021) produced the metagenomes following an annotation methodology (Huntemann et al., 2016) that predicts protein-coding genes. The protein-coding genes are associated with proteins by assigning KEGG orthology terms (KO) (Kanehisa et al., 2016), from the KEGG database (Kanehisa, 2000), using LAST (Kielbasa et al., 2011).

The genes considered for this study are summarized in Table 1 and follow the current data available from the Kyoto Encyclopedia of Genes (KEGG) (Kanehisa, 2000). After selecting the KOs associated with nitrogen-cycling, according to Table 1, the GeneID for the selected KOs was automatically extracted, from all metagenomes, using a Python script. A GeneID is the unique identifier of each sequence, within a given metagenome. Each GeneID has an Estimated Copy value, which is the number of sequences expected. The Estimated Copy value for each GeneID is an output from the JGI IMG/M platform (Supplementary Table 3).

**TABLE 1:** Summary of the nitrogen-cycle pathways available on the Kyoto encyclopedia of genes (KEGG) database.

Protein	Gene	KO code	Metabolic pathways involved
NarGHI/NxrAB	*narG, narZ, nxrA*	K00370	DNR, denitrification and nitrification
	*narH, narY, nxrB*	K00371	
	*narI, narV*	K00374	
NapAB	*napA*	K02567	DNR and denitrification
	*napB*	K02568	
NarB	*narB*	K00367	ANR
Assimilatory nitrate reductase (NR)	NR	K10534	ANR
NasAB	*nasA*	K00372	ANR
	*nasB*	K00360	
NirBD	*nirB*	K00362	ANR and DNR
	*nirD*	K00363	
NrfAH	*nrfA*	K03385	DNR
	*nrfH*	K15876	
Nitrite reductase (NIT-6)	NIT-6	K17877	ANR
NirA	*nirA*	K00366	ANR
NirK	*nirK*	K00368	Denitrification and Anammox
NirS	*nirS*	K15864	
NorBC	*norB*	K04561	Denitrification
	*norC*	K02305	
NosZ	*nosZ*	K00376	Denitrification
NifDKH	*nifD*	K02586	Nitrogen fixation
	*nifK*	K02591	
	*nifH*	K02588	
AnfG	*anfG*	K00531	Nitrogen fixation
VnfDKGH	*vnfD*	K22896	Nitrogen fixation
	*vnfK*	K22897	
	*vnfG*	K22898	
	*vnfH*	K22899	
AmoCAB/MMO	*pmoA-amoA*	K10944	Nitrification
	*pmoB-amoB*	K10945	
	*pmoC-amoC*	K10946	
Hao	*hao*	K10535	Nitrification
Hzs	*hzsC*	K20932	Anammox
	*hzsB*	K20933	
	*hzsA*	K20934	
Hdh	*hdh*	K20935	Anammox

### Statistical analysis

For each gene in Table 1, and for each sample, three variables were calculated: Gene abundance, which is the number of sequences of a gene, computed as the sum of the Estimated Copy value of each GeneID associated with the respective KO identification; Gene richness, which is the number of different sequences of a specific gene; Gene variance, which is the variance of the Estimated Copy of all GeneIDs, that are associated with a respective KO. The selected variables from Supplementary Table 2 were renamed for simplicity in Supplementary Table 4.

To help grasp and visualize the relations between the presence and absence of different nitrogen-cycling genes, or between nitrogen-cycling genes and biogeochemical variables, the correlation between these variables was calculated using the Pearson coefficient. This coefficient measures linear correlation between two variables and can take values between -1 and 1, representing negative or positive correlation, respectively, and with 0 meaning the absence of any linear correlation. This statistic was then represented using the corrplot R-package (Wei et al., 2021), for the cases with statistical significance (*p* < 0.05).

Association Rules (ARs) were used to discover more complex and novel patterns/relationships between the distribution of nitrogen-cycling genes and the multiple biological and/or biogeochemical variables. ARs are logical assertions describing a relationship between an antecedent and a consequent. Both consist of a set of categorical items (qualitative variable) and indicate that whenever the antecedent happens, the consequent is likely to occur (AR: if antecedent, then consequent). The strength of this relationship is characterized by some statistical measures, the most frequent being support, lift and confidence as described below. Because ARs only work with categorical items and most of the metadata under analysis was quantitative, the quantitative variables were grouped into qualitative variables. All variables were discretized based on two (0–50%, 50–100%) or three categories (0–30%, 30–70%, 70–100%), meaning that the lower values of the variables are represented by the categories “<30%” or “<50%”, while the higher values are represented by “>70%” or “>50%.” The partitioning values can be seen in Supplementary Table 4.

The ARs were obtained using the *Apriori* algorithm (Agrawal and Srikant, 1994), implemented in the arules R-package (Hahsler et al., 2011). These were evaluated using a set of standard statistics that quantify the quality of the rules. The Support measures how frequent is the simultaneous occurrence of the antecedent and the consequent as a percentage from all the dataset. For example, for an AR: {Fungi phylotypes ≥ 70%} = > {NxrAB = Present}, a support of 0.22 means that in 22% of the metagenomes in analysis, Fungi phylotypes ≥ 70% and NxrAB = Present is true; The Confidence measures the probability of the consequent to occur whenever the antecedent occurs. For example, for an AR: {Fungi phylotypes ≥ 70%} = > {NxrAB = Present}, a confidence of 1 means that in 100% of the cases where Fungi phylotypes is above 70%, NxrAB is present; the Lift accounts also for the frequency of the consequent, by dividing the confidence of the rule by the expected confidence (probability of occurrence of the consequent), if equal to 1 both antecedent and consequent are independent. Higher values of Lift mean that there is indeed a very strong dependence of the consequent on the antecedent, i.e., if the antecedent is true then there is a very strong probability that the consequent will also be true. Importantly, if a single gene from the operon was found, but not the other genes, we considered the respective protein to be absent (recall that we can only refer to the potential to express the protein, not the actual expression).

### Data visualization

To visualize the genetic richness and abundance of nitrogen-cycling genes across all metagenomes, the Circos software was used (Krzywinski et al., 2009). In this visual representation, the genes were grouped into proteins, which were grouped by pathways, allowing a quick insight on the genetic diversity of the nitrogen cycle in MDV.

A map localizing the sampling points was obtained in QGis (QGIS Development Team, 2016), using Quantarctica data (Matsuoka et al., 2018). Further mapping visualizes the spatial abundance, richness, and variance of the genes from Table 1, and was produced using ggplot2 (Wickham, 2016), raster (Hijmans, 2020), and rgdal (Bivand et al., 2022) R-packages, in the R work environment (R Core Team, 2020). For the purpose of visualizing the spatial distribution of relevant environmental variables in the map, the variables of percentage of water in soil, pH, biomass, and percentage of nitrogen were computed using Akima’s algorithm for linear interpolation (Akima, 1978), since the data are irregularly spaced. The calculation was performed using R-package akima (Akima et al., 2022) and visualized with ggplot2 (Wickham, 2016) in R work environment (R Core Team, 2020).

## Results

### Nitrogen-cycling genes frequency and interrelationships in McMurdo Dry Valleys

**FIGURE 2:**
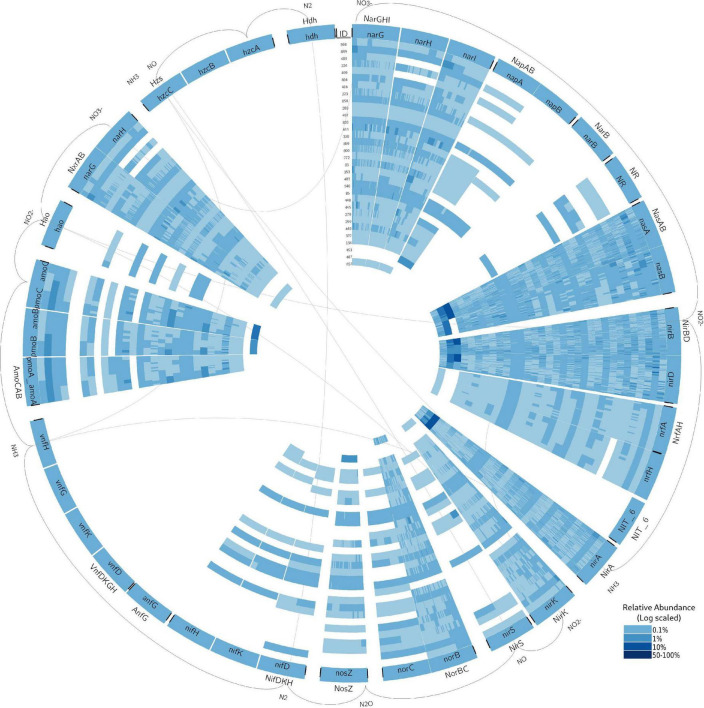
Circular visualization of all nitrogen-cycling genes richness and relative abundance. Read the figure clockwise, from the exterior of the circle to the interior. The nitrogen-cycle reactions (based on Table 1) are illustrated by the chemicals linked with black external links. Within each link, all proteins that can perform those reactions are included. The proteins are within bold black ticks and divided into blocks, each block relative to the gene encoding a subunit of the protein. For example, NarGHI protein is divided in three blocks, for the genes *narG*, *narH*, and *narI*. Internal light gray links connect the molecules that are repeated. The 32 metagenomes are plotted as heatmaps, with respective unique codes. Each heatmap was divided by the number of different sequences where the gene (of the specified block) was found. The color is proportional to the relative abundance (scale log_0_._05_).

Results from this study showed that the relative abundance and richness of the nitrogen-cycling genes involved in the nitrogen biogeochemistry are highly variable in the different MDV soil metagenomes (Figure 2). Overall, the more abundant and rich nitrogen-cycling genes (e.g., *narGHI*) presented a wider spatial distribution, compared with the less abundant nitrogen-cycling genes (e.g., *hzs*), which were infrequently identified in the study area (Figure 2). Interestingly, all genes belonging to the same operons did not necessarily follow the same pattern of distribution (e.g., *norBC*). Below we provide a detailed description of the results obtained for nitrogen-cycling genes distribution in the metagenomes surveyed, for each nitrogen-cycling pathway.

**FIGURE 3:**
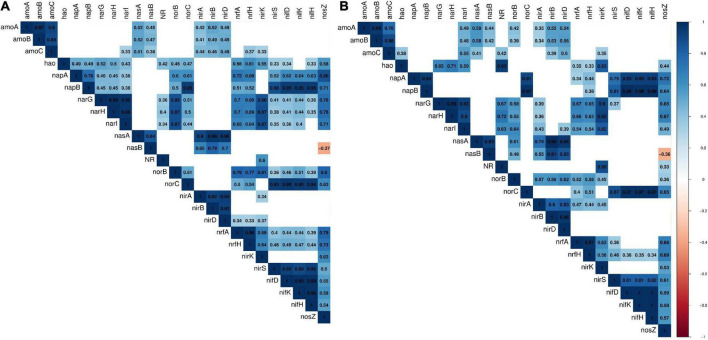
Gene with gene correlation plots panel **(A)** is for gene richness and panel **(B)** is for gene abundance. The red gradient indicates negative correlation, while the blue gradient indicates positive correlation (up to + 1). Non-significant (*p*-value > 0.05) correlations were not illustrated.

For nitrogen fixation, *nifDKH* were fully identified in 21.9% of sites (Figure 2). The presence of *nifDKH* genes coincides with sites where the denitrification genes *nosZ* and *norBC* were present, coherent with the positive correlation (Pearson correlation) between the richness of the genes involved in both pathways, which was highest for *norC* (Pearson coefficient ranged from 0.54 to 0.59 for *nosZ*; from 0.39 to 0.51 for *norB*; and from 0.94 to 0.95 for *norC*, Figure 3A). The same pattern was found for the abundance of those genes, except for *norB* gene, with a non-significant correlation, while *norC* gene had a 0.97 positive correlation with *nifDKH* (Figure 3B).

For nitrification, *amoCAB* were identified in two-thirds of all sites, with most sites presenting low richness (Figure 2). Association rules found *amoCAB* genes to be related with the presence of denitrification and DNR-associated genes (Supplementary Table 5). Further in nitrification, *hao* was infrequently found (15.6% of sites, with low gene richness and abundance, Figure 2) and the richness, abundance, and frequency of the genes involved in the nitrite oxidation (*nxrAB*) were higher than the remaining genes implicated in nitrification (Figure 2). Be noted that *nxrAB* and *narGHI* genes also contribute to DNR and denitrification pathways (Table 1). These genes were positively correlated with *hao* richness (ranged from 0.43 to 0.52, Figure 3A) and abundance (ranged from 0.59 to 0.71, Figure 3B).

Comparing denitrification, DNR and ANR, gene richness was lower for genes involved in denitrification and DNR (*narGHI*), but gene abundance was higher for *nasAB* (involved in ANR). Further, *nasAB* was ubiquitous and diverse (identified in 96.8–100% of sites). Other genes associated with nitrate reduction (ANR and DNR) were less prevalent. For example, *napA* was identified in 59.4% of sites and *napB* in 15.6% of sites. Moreover, *narB* was absent in any sites and nitrate reductase gene (NR) was identified in 18.8% of sites (Figure 2).

For the nitrite reduction step in denitrification, *nirK* was present in most sites (96.9% of sites); however, *nirS* was only found in 34.4% of samples (Figure 2). The presence of *nirK* was correlated with genes from all the pathways of nitrogen-cycling (Supplementary Table 5). Further on, the nitric oxide reductase gene *norB* had a higher richness, abundance, and frequency (present in 93.8% of sites) than *norC* (53.1% of sites). In addition, *nirS* and *norBC* genes’ relative abundance was significantly correlated with the genes involved in nitrous oxide reduction step (*nosZ*) (from 0.5 to 0.8, Figure 3B). The only significant negative correlation identified within all nitrogen-cycling genes interrelationships was between the richness and abundance of *nosZ* and the assimilatory nitrate reduction *nasB* gene (Figure 3).

For the dissimilatory nitrate reduction pathway, gene richness was higher for *nirBD* than *nrfAH* (Figure 2), and both were identified in all sites. Similarly, the *nirA* gene associated with ANR was identified in all sites and with high richness in most of the sites (Figure 2). Comparing the richness of ANR genes for the same pathway, *nirA* richness was correlated with *nirB* (0.92, Figure 3A) and *nirD* (0.94, Figure 3A). Furthermore, *nirA* relative abundance was positively correlated with *nrfAH* relative abundance (ranged from 0.36 to 0.47, Figure 3B), but not significantly correlated with its richness (Figure 3A). Comparing *nirA* with *narGHI* genes (involved in nitrate reduction to nitrite), there was no significant correlation for the richness, but there was for abundance (ranged from 0.36 to 0.43, Figure 3B). The *nasAB* genes were associated with the presence of genes for several pathways, including *nrfAH* genes for DNR and all genes for denitrification. The *nasAB* genes were also associated with the presence of *amoCAB* and *nifDKH* (Supplementary Table 5).

Overall, the gene richness (Figure 3A) and abundance (Figure 3B) correlations between the different genes involved in the nitrogen-cycling pathways were positive. This pattern was supported by the association rules identified (Supplementary Table 5), showing that genes encoding enzymes from specific nitrogen-cycling pathways were usually positively associated with each other.

### Spatial distribution of nitrogen-cycling genes in McMurdo Dry Valleys

**FIGURE 4:**
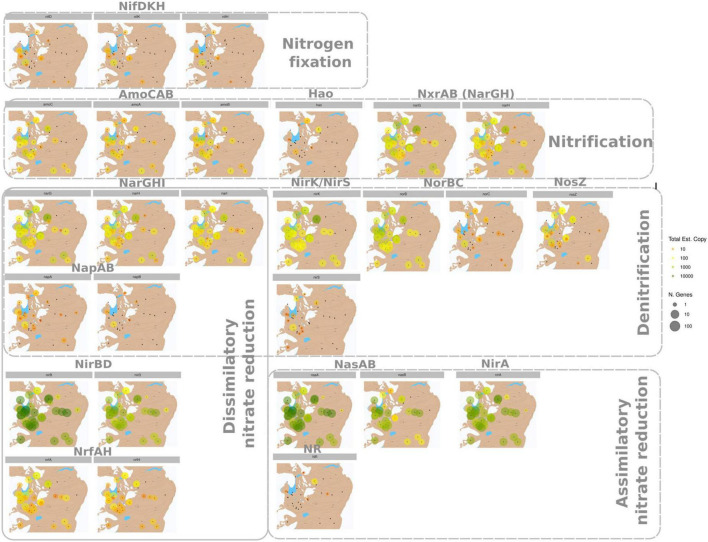
Mapping of gene abundance and richness grouped by pathway and ordered by protein and reaction order. For each of the analyzed genes from Table 1 that were identified the total estimated copy number (which indicates gene abundance) is illustrated with a color gradient from orange (lowest) to green (highest); The number of genes is illustrated with the size of the site-points, from lowest number of genes to highest number of genes and indicate gene richness.

The soil samples used in this study for the spatial analysis were distributed across several kilometers, between the Miers valley, the Garwood valley, and Joyce glacier (Figure 1), with several samples nearby lake Budha and others scattered throughout the valleys. Below we detail the distribution of the genes implicated in each nitrogen-cycling pathway (Figure 4).

For nitrogen fixation, the samples that showed *nifDKH* with higher richness and abundance were clustered near the Lake Budha (Figure 4) and gene variance was found to be higher between lake Budha and Miers lake (Supplementary Figure 1). Two sites between Findlay Ridge and Becker Point also showed the presence of *nifDKH*, but with lower richness and abundance (Figure 4).

Ammonia monooxygenase genes (*amoCAB*) were present in all stations, with higher richness and abundance near the Lake Budha and Miers Lake (Figure 4) and similarly for gene variance (Supplementary Figure 1). Following the nitrification path, the few samples for which the hydroxylamine dehydrogenase gene (*hao*) was identified were located north of Lake Budha, between the Marshall Ridge and Garwood Glacial (Figure 4), where *hao* gene variance was also higher (Supplementary Figure 1).

Genes for complete denitrification were identified throughout the sampling region. The genes involved in nitrate reduction (*narGHI*) were present everywhere (Figure 4), with high gene variance (Supplementary Figure 1). Results showed that *nirK*-type denitrifiers are more spatially dispersed than *nirS*-type denitrifiers, and latter one had richness and abundance concentrated north of lake Budha, near the Collen lake and Marshall ridge (Figure 4), with low gene variance (Supplementary Figure 1). The *norBC* were dispersed everywhere, but *norC* had lower gene richness, abundance and gene variance than *norB* (Figure 4 and Supplementary Figure 1). The gene involved in the final step of denitrification (*nosZ*) was also dispersed across distant locations, although the samples located north of Lake Budha presented higher gene richness, abundance, and variance (Figure 4 and Supplementary Figure 1).

For DNR, the *napAB* genes were differently distributed: while *napA* was distributed across the entire map, *napB* was constrained between Lake Budha, Garwood glacier, and Rivard glacier. Both genes presented low abundance and low richness (Figure 4), and *napB* showed less gene variance than *napA* (Supplementary Figure 1). Genes *nirBD* presented high abundance (Figure 4) and variance (Supplementary Figure 1) for all sites. The *nrfAH* genes were dispersed everywhere, with lower abundance, richness, and genes variance than *nirBD* (Figure 4 and Supplementary Figure 1).

For ANR, NR genes presented higher abundance in the Marshal ridge area and lower in the areas of lake Buddha, Rivard Glaciar, and Marsal Ridge. The *nasAB* genes were widely distributed, with high richness, abundance (Figure 4), and gene variance (Supplementary Figure 1). Finally, *nirA* gene was also broadly distributed with high richness, abundance (Figure 4), and high gene variance (Supplementary Figure 1).

Overall, the nitrogen-cycling genes with an ubiquitous distribution across all sites were also the most abundant genes, and the less abundant genes were more concentrated near Lake Budha and Miers lake (Figure 4 and Supplementary Figure 1). We did not find a clear relationship between the location and the presence of specific nitrogen-cycle pathways, results suggested that as long as a gene was very abundant, it was widespread to all surveyed regions. Furthermore, the gene variance was generally similar for genes implicated in the same pathway and the sites with higher gene richness and abundance resulted in higher gene variance.

### McMurdo Dry Valleys biogeochemistry controls on the distribution of nitrogen-cycling genes

**FIGURE 5:**
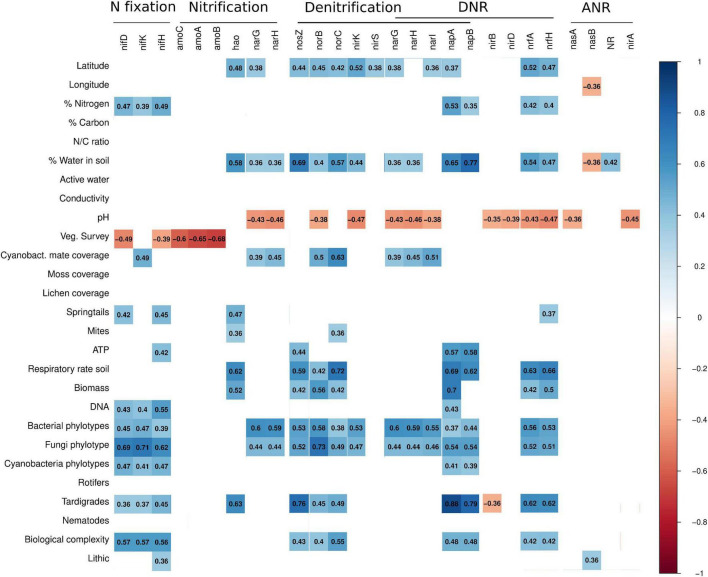
Gene richness with biogeochemical variables correlation. The red gradient indicates negative correlation **(down** to -1), while the blue gradient indicates positive correlation **(up** to +1). Non-significant (*p* > 0.05) correlations were not illustrated.

**FIGURE 6:**
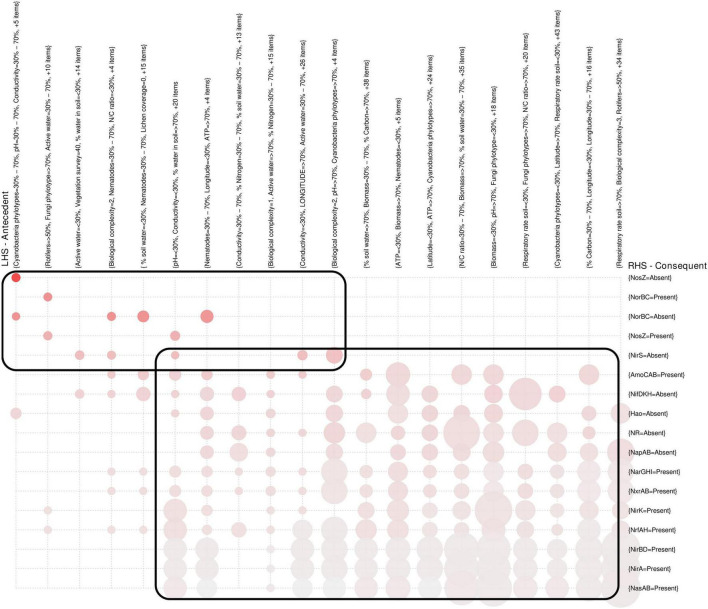
Association rules between presence/absence of operons and biogeochemical variables. Illustration of a clustering of the most significant association rules (*n* = 676), with size as support, color as lift and consequent/antecedent of the association rules specified in the text, with lines as presence/absence of the entire operons and columns as association rules for those operons. More specifically, the columns describe the left hand side (LHS), or antecedent, and the lines describe the right hand side (RHS), or consequent. For example, for the presence of operons for NorBC there was a clustering with association rules corresponding to the environmental variables of Rotifers (with values superior to 50% of samples), fungi phylotype (with values superior to 70% of samples), active water (with values within the range of 30–70% of samples) and 10 other variables that were omitted due to visualization constraints (indicated as “+10 items”). The full description of the association rules clustered in this figure are available in Supplementary Table 6.

Correlations between the selected biogeochemical variables and both richness and abundance of nitrogen-cycling genes presented similar patterns (Figure 5 and Supplementary Figure 2), but overall correlations were stronger for gene richness. The only environmental variables that presented significant and negative correlations across nitrogen-cycling genes from all pathways were pH and vegetation (Figure 5 and Supplementary Figure 2). Biological factors, like taxa richness [a measure of biological complexity from Lee et al. (2019)], and abiotic factors, like soil water content, were significantly positively correlated with most nitrogen-cycling genes (Figure 5 and Supplementary Figure 2). Some genes, like *amoCAB*, *nirBD*, *nirA*, and NR, were correlated with fewer environmental variables. For example, *amoCAB* only presented negative correlations with vegetation [area covered by vegetation of moss, lichen and cyanobacterial mats from Lee et al. (2019)] (Figure 5 and Supplementary Figure 2). Further, we did an AR analysis between biogeochemical variables and the nitrogen-cycling genes (Figure 6 and Supplementary Table 6), which were grouped by the operons responsible for the different subunits of the protein they encode for. From this analysis, two general groups were distinguished, one corresponding to the *nirS*, *norBC*, and *nosZ* genes (denitrification), and the other group corresponds to the remaining pathways (for DNR, ANR, nitrification, and nitrogen fixation). A more detailed description on the observed relationships between the different nitrogen-cycling pathways and environmental parameters is given below.

The most relevant environmental variables related with the distribution of nitrogen-fixation genes (*nifDKH*) were the ones that are indicators of biological diversity (e.g., taxa richness, bacterial diversity, fungal diversity, and cyanobacterial coverage) and nitrogen content (Figure 5 and Supplementary Figure 2). In agreement, AR analysis revealed that the absence of *nifDKH* is associated with lower values or an absence of several biological variables like lichen, moss, and cyanobacterial coverage as well as tardigrades, springtails, and rotifers abundance (Figure 6 and Supplementary Table 6).

For nitrification, besides the significant negative correlation between *amoCAB* genes and vegetation (Figure 4 and Supplementary Figure 2), several association rules with strong confidence level associate the presence of *amoCAB* genes with high percentage of carbon (>70%) and nitrogen (>70%) as well as high biomass in the soil sample (>70%) (Figure 6 and Supplementary Table 6). The richness of *hao* gene presented positive correlations with many biological and biogeochemical variables, including, for example, the number of tardigrades, mites, springtails, and soil water content (Figure 5), but the abundance of *hao* is only significantly and positively correlated with soil water content (Supplementary Figure 2).

The richness of genes associated with nitrite oxidation or nitrate reduction, such as *nxrAB/narGHI* (Table 1), was positively correlated with bacterial and fungal diversity and cyanobacteria coverage and negatively correlated with pH (Figure 5). NR and *napAB* were positively correlated with soil water content (Figure 5). Furthermore, the *napAB* genes were positively correlated with several biological variables (e.g., bacterial diversity and ATP concentration) (Figure 5 and Supplementary Figure 2).

Following the nitrite reduction in the denitrification pathway, the *nirK* gene richness was positively correlated with few abiotic and biotic variables, like soil water content, bacterial diversity, estimated number of bacterial and fungal phylotypes, nitrogen concentration in soils, and negatively correlated with pH (Figure 5). However, the abundance of *nirK* was only correlated positively with soil water content and negatively correlated with pH and DNA concentration (Supplementary Figure 2). *nirS* occurred very infrequently in MDV without clear relationships with the environmental variables.

The *norBC* gene richness was positively correlated with several biological variables (e.g., soil respiratory rate, biomass) and soil water content, but negatively correlated with pH (Figure 5). However, the abundance for *norBC* genes presented very few correlations, for instance, *norC* abundance only correlated positively with soil water and *norB* only correlated negatively with pH and vegetation survey (Supplementary Figure 2). Finally, the *nosZ* gene was positively correlated with soil water content, for both gene richness and abundance (Figure 5 and Supplementary Figure 2). For the gene richness, there were more biological variables positively correlated (e.g., ATP concentration, soil respiratory rate, biomass). The presence and absence of *norBC* and *nosZ* genes in soil samples are associated with a distinct pattern of association rules comparing with the other nitrogen-cycling genes (Figure 6 and Supplementary Table 6) suggesting that nitric oxide and nitrous oxide reduction pathways are associated to a different combination of environmental variables in MDV.

For the ANR pathway, *nirB* abundance was negatively correlated with soil water content (-0.36, Supplementary Figure 2) and *nirBD* abundance was negatively correlated with vegetation survey (from -0.36 to -0.41, Supplementary Figure 2). The genes *nrfAH* were widely distributed and their richness was significantly and positively correlated with most biological variables, and negatively correlated with pH. For the DNR pathway, the *nirA* gene richness was only significantly and negatively correlated with pH (Figure 5), although association rules associated the presence of this gene with several biological variables (Figure 6 and Supplementary Table 6).

Overall, our results suggested that the genetic potential that captures nitrogen and recycles it from nitrogen to nitrates and from nitrates to ammonia is associated with different variables than those for the reactions that remove nitric oxide back to the atmosphere as molecular nitrogen (last steps of denitrification). Besides the two major groups seen in Figure 6, the existence of hundreds of rules complicates the analysis of the specific variables determining the associations, but indicates that complex combinations of biogeochemical variables were relevant in explaining in some way the nitrogen cycle pathways distribution in MDV.

## Discussion

In this study, we hypothesized that the conditions in the soils from MDV pose limits on the nitrogen-cycling pathways and this assumption was overall supported by our metagenomics survey and available metadata. In fact, the spatial distribution of nitrogen-cycling genes was coherent with the linear and non-linear correlations between genes prevalence and biogeochemical variables, because the operons for most nitrogen-cycling genes were present where abiotic factors were less extreme (higher water availability, less alkaline pH) and biotic factors more prevalent (e.g., higher microbial diversity and tardigrades presence). For the soils that were sampled far from lakes and glaciers, the diversity of nitrogen-cycling genes was much lower. Overall, this is consistent with previous studies that used similar methods for a global soil perspective of the nitrogen-cycling genes diversity, where it was found that soils from tropical and human-associated environments (non-extreme environments) present higher richness of nitrogen-cycling genes, while the polar deserts present the scarcest (Fierer et al., 2012; Nelson et al., 2016). Furthermore, previous studies on MDV soils and rocks have already found that these environments harbor genes representative of the entire nitrogen cycle, as reviewed elsewhere (Chan et al., 2013; Wei et al., 2016; Ortiz et al., 2020). Most studies have focused on amplicon sequencing of well-known, conserved marker genes, most notably *nifH* for nitrogen fixation (e.g., Coyne et al., 2020) or *amoA* for nitrification (e.g., Magalhães et al., 2014; Monteiro et al., 2020; Ortiz et al., 2020). Thus, previous studies based on marker gene approach have also collected evidence for the presence of several nitrogen-cycling genes and respective pathways.

**FIGURE 7:**
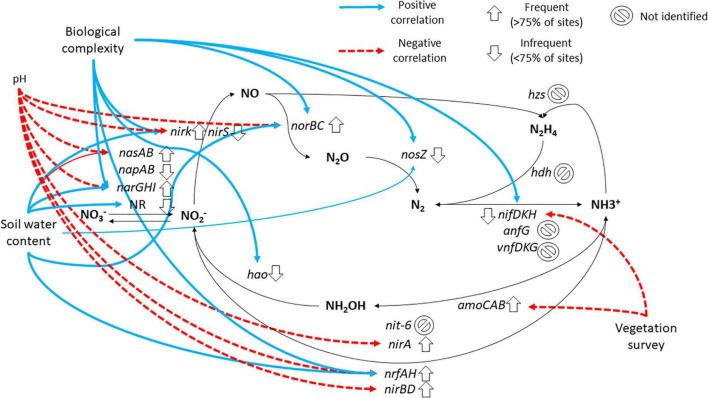
Schematic representation of nitrogen cycle, with implicated genes and summary of relevant biogeochemical variables. Chemical compounds in bold. Black arrows indicate the direction of the reactions of oxidation and reduction. The up arrows indicate that at least one gene of the operon was identified in at least 75% of sites, while the down arrows indicate the opposite (identified in less than 75% of sites). Genes that were not identified in any metagenome have a circular sign. Important correlations and associations between biogeochemical variables (biological complexity, pH, soil water content, vegetation survey) and specific genes were depicted with blue with continuous line **(positive)** and red with discontinuous line **(negative)** arrows. Reactions and genes based on Table 1.

We summarized our metagenomic *vs.* metadata “big picture” of nitrogen-cycling genes of MDV in Figure 7. Anammox was the only nitrogen-cycling pathway not identified in our metagenomic survey, contrary to other MDV functional surveys based on GeoChip microarrays that reported the presence of anammox genes (Chan et al., 2013; Wei et al., 2016). While the utilization of GeoChip microarray allowed for a genetic view of the entire nitrogen biogeochemical cycle (Chan et al., 2013; Wei et al., 2016), the discovery of nitrogen-cycling gene diversity and operons requires metagenomic strategies like it was used in this study. Possible reasons for the absence of the full anammox genetic pathway in our study are insufficient sequencing power to capture rare genes, thus we cannot prove the absence of anammox genes using only the metagenomics approach.

For denitrification, we also found different patterns from previous literature (Jung et al., 2011), specifically for the nitrite reduction step. We found *nirS* to be considerably less abundant and less frequent than *nirK*. This difference might be because in the previous study where these genes were evaluated the authors incubated soil samples from the MDV under different nitrogen content and temperature conditions (Jung et al., 2011), while we surveyed the genes in their original conditions. A study that also surveyed genes in their original conditions, in Antarctic Peninsula, identified all denitrification genes with microarrays, including *nir* family genes, but without distinguishing patterns for *nirK* and *nirS* individually (Yergeau et al., 2007).

Independently of location and other environmental conditions, most genes from all nitrogen-cycling genetic pathways were positively correlated with each other in MDV soils, which is similar to the findings from a global scale survey of the frequency of genes implicated in the nitrogen-cycling genes in soils (Nelson et al., 2016). This suggests that the identification of one nitrogen genetic pathway hints at the presence of the other pathways and that the co-occurrence of genes implicated in the nitrogen-cycling pathways still holds for the microbial communities under the extreme and isolated environment of MDV.

Considering both the spatial position and environmental variables (biotic and abiotic), it was clear that genes with more richness and abundance presented a broad distribution over the sampling area, while the nitrogen-cycling genes with lower diversity and abundance were concentrated in areas with less extreme conditions, near lakes and glaciers. This finding aligns well with previous literature on the relationship between microbial complexity with water and nutrient availability (Niederberger et al., 2012; Kohler et al., 2016; Lee et al., 2018) or pH (Lauber et al., 2009; Fierer et al., 2012). The reason why pH was negatively correlated with nitrogen-cycling genes richness and abundance was probably because of the very alkaline soils of MDV (from 7.49 to 9.67, Supplementary Table 4). Very alkaline soils have been shown to alter the microbial composition in similar soil environments (Lauber et al., 2009) and, consequently, this effect might also decrease the diversity of microorganisms implicated in nitrogen-cycling pathways, lowering the score of richness and abundance of nitrogen-cycling genes with increasing pH. However, it is not possible for us to know, if the negative correlations between pH and nitrogen-cycling genes are due to a direct effect of pH on the actual genes (e.g., gene expression, reaction rates) or if it is a consequence of decreased microbial diversity. pH has been previously identified as a good predictor for differences in microbial biogeographic diversity and richness (Fierer et al., 2012). This study found a positive and significant relationships between soil bacterial diversity and pH, although in this survey soils with pH levels > 8.5 were rare. Most probably there is optimal conditions at near-neutral pH to promote high microbial diversity and richness values in MDV.

Other MDV soil studies (Lee et al., 2018; George et al., 2021) did not find pH as relevant factor in controlling soil microbial community diversity, although the influence of pH in specific metabolic functions, like nitrogen biogeochemistry pathways, has not been evaluated.

The correlation analysis performed supports the view that nitrogen fixation in MDV is influenced by biological interactions, such as fungal diversity (Yergeau et al., 2007) and cyanobacteria (Jungblut and Neilan, 2010), the latter probably because of the capacity for autotrophic nitrogen fixation. Contrary to this, the only variable that we found to be significantly correlated with the ammonia monooxygenase genes (*amoCAB*) was vegetation, mostly lichen and moss (negative correlation). An inverse relation between grass coverage and the dominant AOA ammonia oxidizers has been previously described for savanna soils (Srikanthasamy et al., 2022). Competition for ammonia and decrease in soil aeration in vegetation-covered areas may explain the lower frequency of nitrification genes in these regions, since nitrification is dependent on oxygen concentrations and ammonia availability (Wendeborn, 2020).

Amplicon sequencing studies of *amoA* have suggested that AOA and AOB are specific to different regions, which might reflect niche speciation according to soil type (Magalhães et al., 2014; Monteiro et al., 2020). If that is the case, then soil geochemistry could be an environmental control for nitrifiers. In fact, a study on nitrification rates from soil microbial communities from Miers and Beacon valleys did show different rates of nitrification for each soil (Monteiro et al., 2020). Miers Valley, where the environmental conditions are milder, harbor ammonia-oxidizing microorganisms potentially more active and more abundant than those found in Beacon Valley with more extreme environmental conditions (Magalhães et al., 2014; Monteiro et al., 2020).

It is worth noting that most probably there are very complex combinations of environmental interactions with different microorganisms that result in the survival of the entire nitrogen cycle in these environments. This view was supported by our AR analysis, where we were able to select several strong non-linear correlations between the presence of entire operons and complex combinations of biogeochemical conditions (with rules composed of up to hundreds of different biogeochemical variables). As far as we know, this was the first study using association rules to find these very complex interactions, and allowed us to identify a clear distinct pattern of environmental variables interaction for denitrification compared with the remaining nitrogen-cycling pathways. This finding hints at the possibility that there might be fundamentally different environmental mechanisms controlling denitrification, mainly nitrite, nitric oxide, and nitrous oxide reduction that must be further investigated in future studies.

Genes within the same operon were not consistently identified in the same samples. This was the case for *norBC*, *nasAB*, *nifDKH*, *amoCAB*, and *narGH*. These discrepancies might indicate insufficient sequencing power to capture the full genetic richness of our samples. Low biomass in MDV soils (Hogg et al., 2006) can decrease the observed genetic diversity and explain why some genes were never found. Furthermore, the analysis was purely of functional genes, with no taxonomic information, thus, there was no separation between AOA and AOB, for example, which are known to have dichotomic distributions in MDV (Magalhães et al., 2014). Additionally, the presence of genes is not informative of their expression, neither can we know if they belong to dead cells, although relic DNA has not been shown to be problematic in the Antarctic environment so far (Malard et al., 2019). Although we only used metagenomic data, we do recognize the need for the scientific community to go beyond the identification of genes and tackle new *omics* levels (metatranscriptomics, metaproteomics, and metabolomics), as well as novel *in situ* quantification of the actual nitrogen-cycling reactions rate (Ortiz et al., 2020). Furthermore, we also recognize the limitations of using *omics* approaches, for example, we cannot assert whether a specific gene is absent in a sample, we can only assert whether it was identified or not.

In conclusion, despite the hindrances described above, we provide a detailed description of the biogeography of the genes implicated in nitrogen cycling in MDVs and relate their presence and diversity with several environmental variables, as summarized in Figure 7. In summary, genes for all nitrogen-cycle pathways were identified, except for anammox, and were positively correlated with biological complexity and soil water content. A selected group of genes from assimilatory/dissimilatory nitrate reduction and denitrification were negatively correlated with pH and genes from ammonia oxidation and molecular nitrogen fixation were negatively correlated with vegetation survey (Figure 7). The most frequent and abundant genes were *narGHI* and *nasAB*, implicated in nitrate reduction, *nrfAH* and *nirA*, implicated in nitrite reduction, *nirK* and *norBC*, implicated in nitrite and nitric oxide reduction and *amoCAB*, implicated in ammonia oxidation. Other genes were identified, but were less frequent, like *nosZ*, implicated in the final reduction of nitrous oxide to molecular nitrogen and *nifDKH*, involved in the capture of molecular nitrogen into ammonia. The spatial distribution of the nitrogen-cycling genes showed that locations near the glaciers and lakes provide conditions for the full nitrogen-cycling pathways, while other more arid areas only include the most abundant nitrogen-cycling genes representatives of the most relevant nitrogen-cycling pathways.

## Data availability statement

All information relative to biogeochemical variables and spatial coordinates is available in the article by Lee et al. (2019) and the selected metadata used in this study is available in Supplementary Table 2. The metagenomes used are available from the JGI/MGI platform (https://img.jgi.doe.gov/) using the MetagenomeID available in Supplementary Table 1 and all GeneID’s used are available in Supplementary Table 3.

## Author contributions

FP, IA, LT, and CM conceptualized the manuscript. PB wrote the python script to automatically obtain and organize the metagenomes from JGI/MGI platform. IA wrote the R code for all statistical analysis and FP for the Circos figure. FP wrote and IA, LT, and CM reviewed and contributed to all manuscript drafts. CL and SC designed the sampling campaign and were responsible for sample processing and sequencing. SC, CM, and CL funded the work. All authors reviewed, improved, and approved the final version of the manuscript.

## Supplementary material

Supplementary Figure 1 Mapping of gene variance grouped by pathway and ordered by protein and reaction order. For each of the analyzed genes (Table 1) the total gene variance is illustrated with a color gradient from yellow (lowest) to green (highest); The number of genes is illustrated with the size of the site-points, from small (lowest number of genes) to big (highest number of genes).

Supplementary Figure 2 Gene abundance with biogeochemical variables correlation. The red gradient indicates negative correlation **(down** to -1), while the blue gradient indicates positive correlation **(up** to + 1). Non-significant (*p*-value > 0.05) correlations were not illustrated.

Supplementary Table 1 Metagenome sample information. For each metagenome the respective unique code from Lee et al. (2019) was added. The MetaGenomeID is the ID used to have access to the specific metagenome in the JGI IMG/M platform. The remaining columns provide information for the size of the assembled genome, percentage of the assembled genome and sampling coordinates.

Supplementary Table 2 Metadata from Lee et al. (2019), corresponding to the metagenomes used in this study. Both the unique code of each sample and metagenome ID were added. More detailed information on the variable codes are available in Supplementary Data from Lee et al. (2019) (link: https://docs.google.com/document/d/1WcMNIlNhY6GsTiSd3zb14SkwqYQv5VPk/edit?usp=sharing&ouid=104678400154685890581&rtpof=true&sd=true).

Supplementary Table 3 Estimated copy value of each GeneID in each metagenome. The metagenome ID allows to connect with the unique code and respective metadata, while the GeneID allows to retrieve information on that sequence in the JGI IMG/M platform.

Supplementary Table 4 Simplified names of selected variables and partitioning values used in association rules, where applicable.

Supplementary Table 5 Values for association rules between genes and genes. Complete list of association rules identified between the presence/absence of genes with each other. For each association rule the values for support, confidence, lift, and count are provided.

Supplementary Table 6 Values for association rules between operons and selected biogeochemical variables. Complete list of association rules identified between the presence/absence of operons with the selected biogeochemical variables. For each association rule the values for support, confidence, lift and count are provided.
