# Supplementary figures and images for: The spatial distribution and biogeochemical drivers of nitrogen cycle genes in an Antarctic desert
Source: Front Microbiol. 2022 Oct 6;13:927129. doi: 10.3389/fmicb.2022.927129 (PMC9583160; doi:10.3389/fmicb.2022.927129)

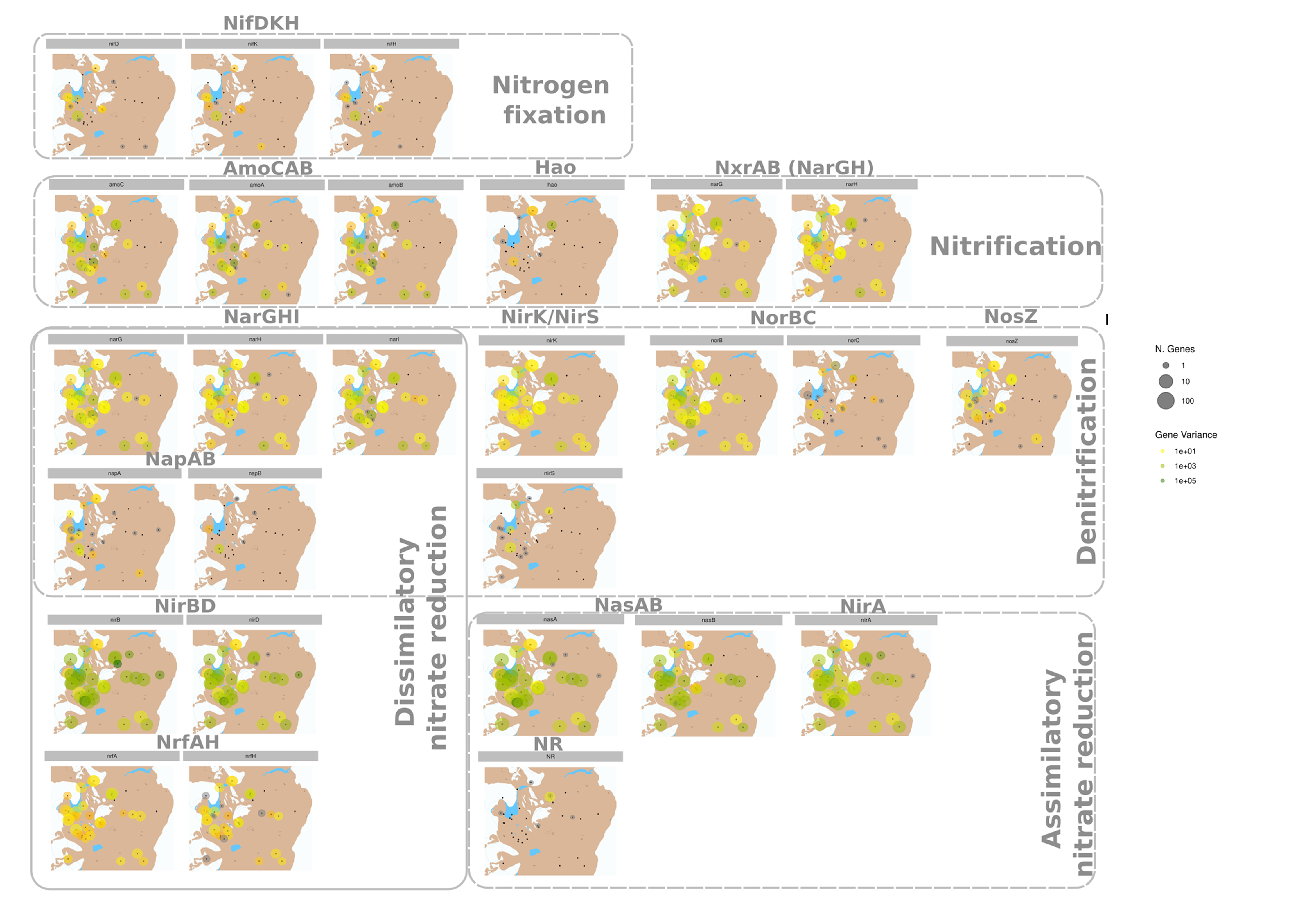

Supplement: Supplementary Figure 1 — Mapping of gene variance grouped by pathway and ordered by protein and reaction order. For each of the analyzed genes (Table 1) the total gene variance is illustrated with a color gradient from yellow (lowest) to green (highest); The number of genes is illustrated with the size of the site-points, from small (lowest number of genes) to big (highest number of genes). [file Image_1.TIFF]

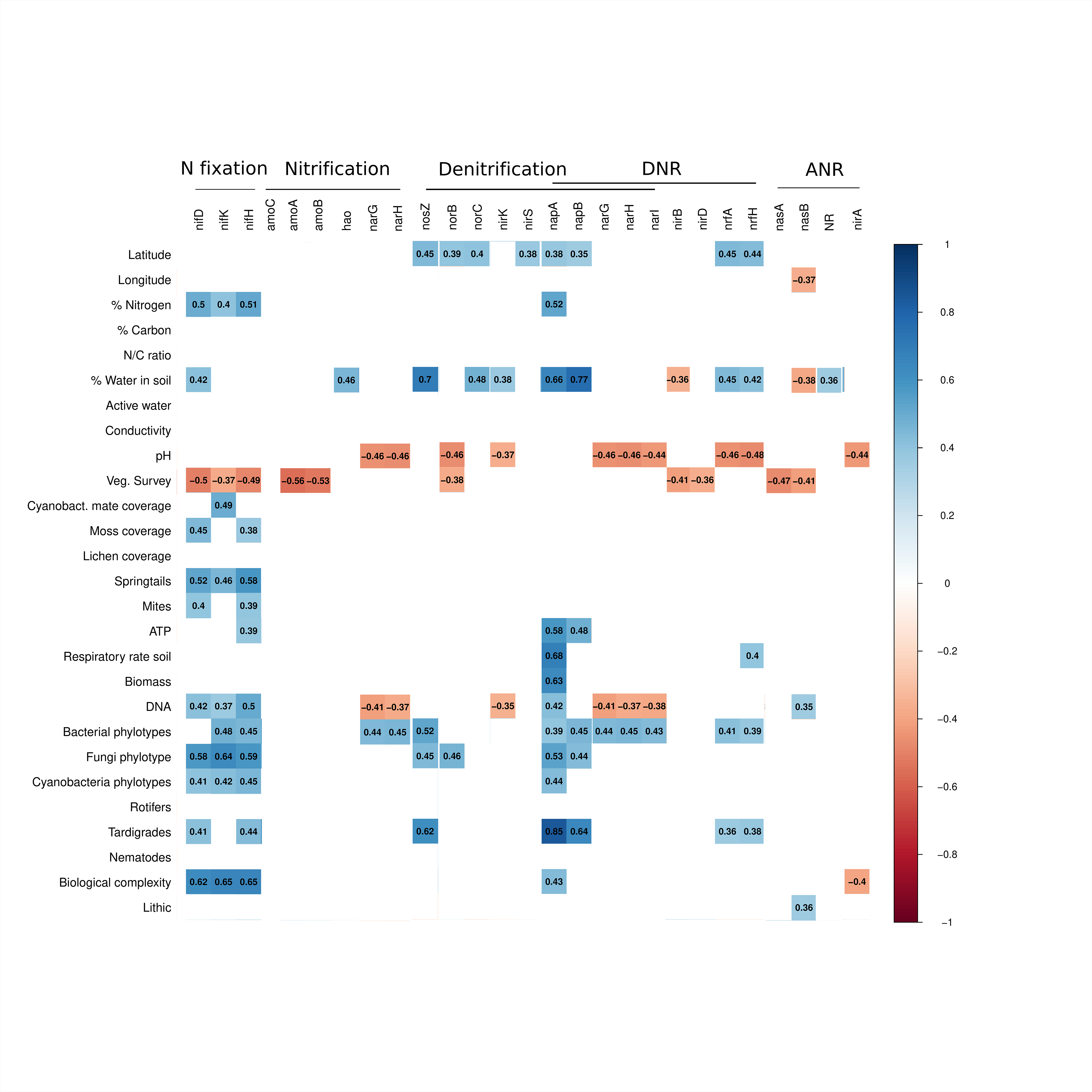

Supplement: Supplementary Figure 2 — Gene abundance with biogeochemical variables correlation. The red gradient indicates negative correlation (down to -1), while the blue gradient indicates positive correlation (up to + 1). Non-significant (p-value > 0.05) correlations were not illustrated. [file Image_2.TIFF]
